# Sphingosine Kinase 1 Plays an Important Role in Atorvastatin-Mediated Anti-Inflammatory Effect against Acute Lung Injury

**DOI:** 10.1155/2021/9247285

**Published:** 2021-12-21

**Authors:** Lan Wu, Yan Cheng, Shunxiang Peng, Wensheng Zhang, Chaoxiong Zhang

**Affiliations:** ^1^Department of Anesthesiology, West China Second University Hospital, Sichuan University, Chengdu, 610041 Sichuan Province, China; ^2^Key Laboratory of Birth Defects and Related Diseases of Women and Children (Sichuan University), Ministry of Education, Chengdu, 610041 Sichuan Province, China; ^3^Department of Anesthesiology, Laboratory of Anesthesia and Critical Care Medicine, Translational Neuroscience Center, West China Hospital, Sichuan University, Chengdu, 610041 Sichuan Province, China; ^4^Department of Center for Disease Prevention and Control, West China School of Public Health and West China Fourth Hospital, Sichuan University, Sichuan, China 610041; ^5^Department of Medicine, University of Illinois College of Medicine, Chicago, Illinois, USA

## Abstract

Atorvastatin is a 3-hydroxy-3-methylglutaryl coenzyme A reductase (HMG-CoA reductase) inhibitor and inhibits cholesterol synthesis. Recently, atorvastatin also showed anti-inflammatory effect in acute lung injury, ameliorating pulmonary gas-blood exchanging function. Sphingosine kinase 1 plays a central role in endothelial (EC) cytoskeleton rearrangement and EC barrier integrity regulation. In this study, the role of sphingosine kinase 1 in atorvastatin anti-inflammatory effect against acute lung injury was investigated. Both wild-type (WT) and SphK1^−/−^ mice were challenged with high tidal volume ventilation (40 ml/kg body weight, 65 breathing/min, 4 hours). The acute lung injury was evaluated and the mechanisms were explored. In WT mice, atorvastatin treatment significantly decreased acute lung injury responding to high tidal volume ventilation (HT), including protein, cellular infiltration, and cytokine releasing; comparing to WT mice, SphK1^−/−^ mice showed significantly worsen pulmonary injuries on HT model. Moreover, the atorvastatin-mediated anti-inflammatory effect was diminished in SphK1^−/−^ mice. To further confirm the role of SphK1 in VILI, we then compared the inflammatory response of endothelial cells that were isolated from WT and SphK1^−/−^ mice to cyclic stretching. Similarly, atorvastatin significantly decreased cytokine generation from WT EC responding to cyclic stretching. Atorvastatin also significantly preserved endothelial junction integrity in WT EC against thrombin challenge. However, the inhibitory effect of atorvastatin on cytokine generation induced by cyclic stretching was abolished on SphK1^−/−^ mice EC. The endothelial junction integrity effects of atorvastatin also diminished on SphK1^−/−^ mouse EC. Signal analysis indicated that atorvastatin inhibited JNK activation induced by cyclic stretch. SphK1 knockout also blocked atorvastatin-mediated VE-cadherin junction enhancement. In summary, by inhibition of MAPK activity and maintenance of EC junction homeostasis, SphK1 plays a critical role in atorvastatin-mediated anti-inflammatory effects in both cellular and in vivo model. This study also offers an insight into mechanical stress-mediated acute lung injury and potential therapy in the future.

## 1. Introduction

Atorvastatin is a member of statin family inhibiting of 3-hydroxy-3-methylglutaryl coenzyme A reductase (HMG-CoA reductase) and modulates cholesterol synthesis [1]. In addition to inhibiting cholesterol synthesis, statins have multiple effects on clinical purposes, such as meliorating the patient's quality of life [2, 3]. Among these properties of statin, the anti-inflammatory effects and enhancement of pulmonary function have been widely investigated [4, 5]. Simvastatin and rosuvastatin have anti-inflammatory effects against LPS and virus infection such as influenza A [6, 7]. Similarly, atorvastatin also attenuated the lung injury and the inflammatory response induced by various stimuli [8, 9].

Ventilation-induced lung injury (VILI) often happens in infant ICU, the patients with coma and in patients with ARDS. The acute lung injury may include the mechanic injury and alkalic metabolic disturbance [10–13]. The pathological basis of VILI includes volutrauma, barotrauma, atelectrauma, biotrauma, and shear strain [14]. The long-term effects include pulmonary dysplasia, mental health issues, and organ dysfunction [15, 16]. The VILI-mediated cellular and molecular pathological changes include endothelial-epithelial barrier dysfunction and increasing endothelial inflammatory responses [17, 18]. Interestingly, atorvastatin demonstrates therapeutic properties against VILI on rabbit isolated lung model, notably the improvement of alveolar capillary permeability and hemodynamics and the attenuation of lung injury induced by high tidal volume mechanical ventilation [4].

Sphingosine kinases are enzymes widely expressed in various tissues, such as brain, intestines, lung, and kidney. Sphingosine kinases include SphK1 and SphK2. Sphingosine kinases are involved in many fundamental cellular processes, including cell survival, proliferation, differentiation, migration, and immune function [19–22]. In addition, SphK1 plays an important role in anti-inflammation with multiple mechanisms on different models [23, 24]. After converting sphingosine into spingosine-1-phosphate (S1P), S1P binds with its receptors and induces downstream effects, such as regulating cellular cytoskeleton arrangement, enhancing endothelial junction integrity, and targeting gene regulation [21, 25–27]. Recently, evidence shows that SphK1 meliorates inflammatory response by modulating MAPK activity, such as downregulation of JNK and P38 activity [28–30]. More interestingly, statin-mediated lung protective effects may be regulated by SphK1 [31]. However, the detail of mechanisms remains unclear.

In present studies, we explored the mechanisms and importance of atorvastatin in high tidal volume ventilation-mediated acute lung injury using a sphingosine kinase 1 knockout mouse model. By regulating the SphK1-MAPK signal pathway, atorvastatin meliorated high tidal volume ventilation-mediated acute lung injury. In addition, atorvastatin also regulates endothelial junction integrity by rearrangement of cytoskeleton system. These findings may offer a new view and potential therapy for the high tidal volume ventilation-mediated acute lung injury.

## 2. Materials and Methods

### 2.1. Materials

The RT-PCR and qPCR kit were purchased from Applied Biosystems (Beverly, MA). The mouse ELISA kits were purchased from BioLegend (San Diego, CA) and Millipore Sigma (Burlington, MA). The CD31-Alexa and CD45-FITC antibodies were purchased from Santa Cruz Biotechnology (Santa Cruz, CA). All other antibodies and reagents were purchased from Cell Signaling Technology (Beverly, MA).

### 2.2. Cell Culture

The isolated mouse primary endothelial cells were cultured in EGM-2 supplemented with 2% FBS, hydrocortisone, hFGF, VEGF, ascorbic acid, hEGF, GA-1000, heparin, and R3-IGF-1 (Lonza, Walkersville, MD). The cells were cultured in 75 cm^2^ flask at 37°C in 5% CO_2_ and 95% air. Once the cells got confluence, the cells were used for experiment or frozen for future usage. Before the cells were used, the purity was checked by flow cytometry and maintained above 90%.

### 2.3. SphK1^−/−^ Mouse Breeding and Models

The C57 black 6 (C57BL6) and SphK1 homozygous knockout (SphK1^−/−^) mice were purchased from Shanghai Animal Model Center, Shanghai, China. SphK1^−/−^ mice were backcrossed with C57BL6 mice for five generations to eliminate the background interference to the observed phenotypes. The siblings of the backcross of the SphK1^−/−^ and C57BL6 mice were used in this study. All experiments and animal care procedures were reviewed and approved by the Institutional Animal Care and Use committee at West China Hospital of Sichuan University (protocol number: 2019292A). The homozygous SphK1^−/−^ mice and the background control mice (WT, 8-12 weeks old) were treated with atorvastatin or vehicle (10 mg/kg body weight overnight, IP) followed by high tidal volume ventilation (40 ml/kg bodyweight, 65 breaths/min, 4 hours). After ventilation, the mice were sacrificed and bronchoalveolar lavage (BAL) was harvested in select animals as previously described [32]. BAL fluid was assessed for cell counts and protein content. The lungs then were used for histological analysis using lung injury score [33].

### 2.4. Mouse Lung Endothelial Cell Isolation and Purification

The SphK1^−/−^ mouse and WT mouse blood in vessel were flushed with 20 ml PBS containing 10 U/ml heparin (Hospira, Lake Forest, IL) via the right ventricle. The mouse lungs were minced and digested in an enzyme cocktail of DMEM containing 1% bovine serum albumin, 2 mg/ml collagenase, 100 *μ*g/ml DNase, and 2.5 mg/ml Dispase at 37°C for 1 hour. The result products were meshed though a 70 *μ*m nylon cell strainer. Once resuspended in 5 ml PBS (containing 1% BSA) and pelleted by centrifuge, the cells were incubated with Fc blocker antibody for 15 min on ice. Then, the cells incubated with anti-CD31 and anti-CD45 for 15 min on ice. After washing with 5 ml PBS (containing 1%BSA), the pulmonary vascular endothelial cells (CD31^+^CD45^−^) were sorted by flow cytometry (BD FACSAria III, Becton, Dickinson and Company, NJ). The CD31^+^CD45^−^ cells from WT mice and SphK1^−/−^ mice were incubated in gelatin-coated plates, and the medium was changed (EGM2 complete medium) next morning [34]. Once setting up the stable cell line, the mRNA of EC was extracted and qPCR was performed to quantitatively determine SphK1 mRNA level in EC. And western blots also were performed to test the protein level in EC.

### 2.5. RT-PCR and qPCR

Once WT cells and SphK1^−/−^ cells were performed experiments, the total RNA was extracted with Qiagen RNeasy Mini Kit (Qiagen, Catalog No. 74104). cDNA was synthetized using High-Capacity cDNA Reverse Transcription Kit (Applied Biosystems, Beverly, MA). Real-time PCR was performed with CFX384 Real-Time System (Bio-Rad Laboratory, Hercules, CA). The standard real-time PCR reaction volume was 20 *μ*l and consisted of 10 *μ*l SYBR Green mix (FastStart Universal SYBR Green Mix kit, Roche, Indianapolis, IN). The initial step at 95°C for 30 seconds and 40 cycles consisted of 10 seconds of melting at 95°C, followed by 30 seconds of annealing/extension at 60°C. All reactions were triplicated. The data is represented as a mean normalized expression by GAPDH [35].

The primers used in real-time PCR are as follows: mouse SphK1: forward 5′-ACAGTGGGCACCTTCTTTC, reverse 5′-CTTCTGCACCAGTGTAGAGGC; mouse GAPDH: forward 5′-CAACTACATGGTCTACATGTTC, reverse 5′-CACCAGTAGACTCCACGAC [36].

### 2.6. Cytokine Measurement in Cellular Medium and in BAL

The endothelial cells isolated from SphK1^−/−^ and WT mice were grown to 80% confluence in Flexcell cyclic stretch plate (Flexcell Tension System, Burlington, NC). After being treated with 5% atorvastatin overnight, the cells were cyclic stretched on Flexcell for 4 hours (18% elongation, 0.5 Hz). After finishing the cyclic stretch, the media were harvested and centrifuged briefly to remove the cellular debris. The supernatants were collected to measure the indicated cytokines. The cytokines in BAL were measured by the same kits that were used to measure the cytokines in cellular medium. The ELISA kits were performed according to the manufacturer's instruction.

### 2.7. Western Blotting

Samples were harvested with cellular lysis buffer containing proteinase inhibitors and phosphatase inhibitors as per standard protocols. After sonication and centrifugation, the supernatant was collected. Laemmli sample buffer was added into the supernatant and then was boiled and subsequently analyzed by SDS–PAGE. After transferring to a nitrocellulose membrane (Bio-Rad, Inc., Hercules, CA), western blotting was performed using appropriate primary antibodies and horseradish peroxidase-conjugated secondary antibodies prior to visualization via chemiluminescence (Amersham Biosciences, Piscataway, NJ). Blot density was determined by Alpha Imager software (Alpha Innotech, San Leandro, CA).

### 2.8. Immunofluorescence Microscopy

Confluent EC grown on coverslips were exposed to 1 U/ml thrombin for 1 hour, fixed with 3.7% formaldehyde, and permeabilized with 0.25% Triton X-100. After blocking with 2% bovine serum albumin for 1 hour, cells were exposed to primary antibodies of interest for 2 hours. Fluorescent-tagged secondary antibodies were applied for 60 min in the dark. Cells were imaged using a Nikon video imaging system [37].

### 2.9. Statistical Analysis

Student's *t*-test was used to compare the means of data from two experimental groups, while significant differences (*P* < 0.05) among multiple group comparisons were performed by one-way ANOVA followed by Tukey's test. Results were expressed as means ± SE.

## 3. Results

### 3.1. Sphingosine Kinase 1 Knockout Diminished the Atorvastatin-Mediated Lung Permeability Enhancement Effect on Murine VILI Model

**Figure 1:**
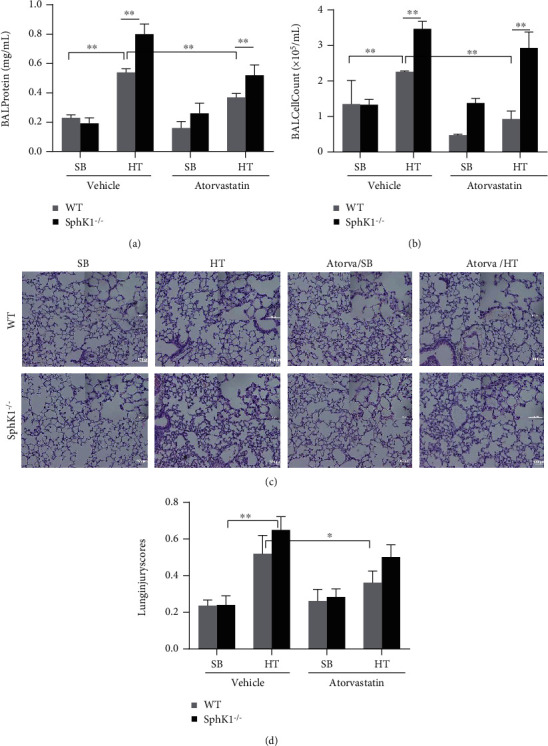
Atorvastatin attenuated VILI-mediated lung permeability but SphK1 knockout reversed this effect. WT and SphK1^−/−^ were treated with atorvastatin overnight as described in Materials and Methods. The next day, the mice underwent high tidal volume ventilation (40 ml/kg, 65 breaths/min, 4 hours). After ventilation, the mouse BAL and lung were harvested for protein, cellular, and histology analysis. (a, b) Compared to spontaneously breathing control mice, HT significantly induced both protein and cells in BAL. Atorvastatin treatment attenuated the HT-mediated induction of protein and cells in BAL. Compared to WT mice, HT-induced SphK1^−/−^ mice had more protein and cell infiltration in the BAL. However, SphK1 knockout significantly abolished the atorvastatin-mediated anti-inflammatory effect in SphK1^−/−^ mice. (c, d) Compared to WT mice, HT-induced SphK1^−/−^ mice had significantly more cell filtration and alveolar structure damage with higher lung injury scores. Moreover, SphK1 knockout reversed atorvastatin-mediated cell filtration inhibitory effect on WT mice (^∗^*P* < 0.05, ^∗∗^*P* < 0.01).

Previous studies indicated that statin had anti-inflammatory effects against various pathogenic agents and SphK1 played an important role of in inflammatory response. We first compared the response of WT and SphK1^−/−^ to high tidal volume ventilation. In Figure 1(a), BAL assay showed that high tidal volume ventilation (HT) induced a significant protein (WT HT vs. WT SB, 0.54 ± 0.025 vs. 0.23 ± 0.02 mg/ml, *P* < 0.01, *n* = 5) and cell filtration (WT HT vs. WT SB, 2.26 ± 0.025 × 10^5^ vs. 1.35 ± 0.67 × 10^5^/ml, *P* < 0.01, *n* = 5) into alveoli compared to spontaneous breathing (SB) in WT mice. Pretreatment of atorvastatin significantly decreased HT-mediated protein and cell filtration in WT mice (WT HT/Atorva, 0.37 ± 0.026 mg/ml and 0.93 ± 0.23 × 10^5^/ml, respectively; *P* < 0.01, *n* = 5).

Compared to WT mice, HT induced significant higher protein leakage (WT HT vs. SphK1^−/−^/HT; 0.54 ± 0.03 vs. 0.80 ± 0.07 mg/ml; *P* < 0.01, *n* = 5) and cell filtration in the BAL of SphK1^−/−^ mice (WT HT vs. SphK1^−/−^/HT; 2.26 ± 0.025 × 10^5^/ml vs. 3.47 ± 0.21 × 10^5^/ml; *P* < 0.01, *n* = 5). More interesting, knockout of SphK1 significantly reversed the inflammatory inhibitory effect of atorvastatin, including protein leakage (SphK1^−/−^ HT/Atorva vs. WT HT/Atorva; 0.52 ± 0.07 mg/ml vs. 0.37 ± 0.03 mg/ml, *P* < 0.01) and cell infiltration in BAL (SphK1^−/−^ HT/Atorva vs. WT HT/Atorva; 2.93 ± 0.45 × 10^5^/ml vs. 0.93 ± 0.23 × 10^5^/ml, *P* < 0.01, respectively, Figures 1(a) and 1(b)).

Histology assay showed that HT induced a mass cell infiltration in alveolar interstitium in WT mice and atorvastatin inhibited this HT-mediated cell infiltration. In contrast, HT induced a higher cell infiltration in alveolar interstitium in SphK1^−/−^ mice and atorvastatin only mildly inhibited this HT-mediated cell infiltration (Figures 1(c) and 1(d)).

### 3.2. Sphingosine Kinase 1 Knockout Abolished the Atorvastatin-Mediated Cytokine Generation Inhibitory Effects against High Tidal Volume Ventilation

**Figure 2:**
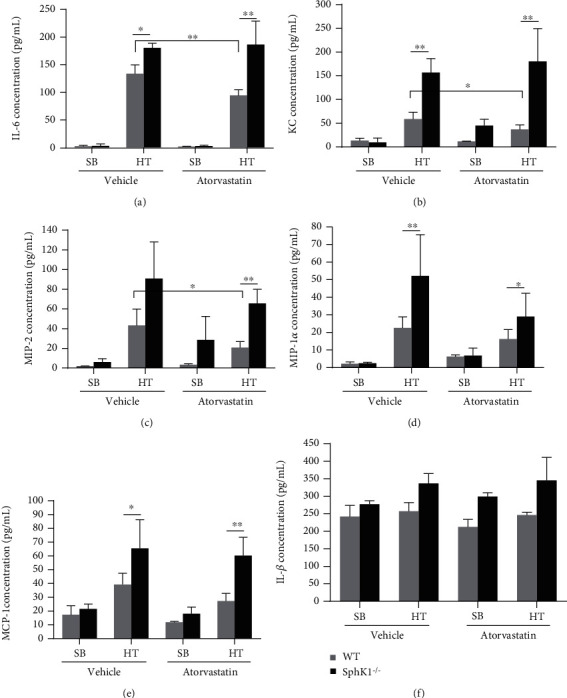
Atorvastatin attenuated HT-mediated cytokine generation in BAL, but SphK1 knockout diminished this effect. ELISA was performed on the BAL harvested from both post-HT WT and SphK1^−/−^ mice as described in Materials and Methods. Similar with protein and cell infiltration, HT significantly increased cytokine release in WT mouse BAL. These cytokines included IL6, KC, MIP-1*α*, MIP-2, and MCP-1, but not IL-1*β*. HT-mediated cytokine release in WT mouse BAL was significantly attenuated by pretreatment of atorvastatin; HT-mediated cytokine generation was significantly higher in SphK1^−/−^ mouse BAL than that in WT mice. Moreover, SphK1 knockout reversed the anti-inflammatory effect of atorvastatin in cytokine generation (^∗^*P* < 0.05, ^∗∗^*P* < 0.01).

Cytokine release from endothelial cells or neutrophil is hallmark of inflammatory response to various stimuli, including high tidal volume ventilation. We then investigated whether sphingosine kinase 1 knockout changed HT-mediated cytokine release on murine model. Similar to HT-mediated protein and cell leakage into mouse alveoli, HT also increased cytokine generation in WT mouse BAL comparing to spontaneous breathing mice, including IL6, KC, MIP-1*α*, MIP-2, and MCP-1, but not IL-1*β*. Atorvastatin pretreatment significantly inhibited IL6 (WT Vehicle/HT vs. WT Atorva/HT, 133.73 ± 16.06 vs. 94.19 ± 10.33 pg/ml, *P* < 0.01, *n* = 5), KC (WT Vehicle/HT vs. WT Atorva/HT, 58.80 ± 14.31 vs. 37.02 ± 9.55 pg/ml, *P* < 0.05, *n* = 5), and MIP-2 (WT Vehicle/HT vs. WT Atorva/HT, 43.32 ± 16.55 vs. 20.67 ± 6.25 pg/ml, *P* < 0.05, *n* = 5) generation responding to high tidal volume ventilation. Atorvastatin pretreatment also showed similar but not significantly inhibitory effect on MIP-1*α* (WT Vehicle/HT vs. WT Atorva/HT, 22.59 ± 6.24 vs. 16.26 ± 5.37 pg/ml, *P* = 0.05, *n* = 5) and MCP-1 (WT Vehicle/HT vs. WT Atorva/HT, 39.26 ± 8.22 vs. 27.26 ± 5.51 pg/ml, *P* > 0.05, *n* = 5) as WT mice responded to high tidal volume ventilation (Figure 2).

Knockout of sphingosine kinase significantly increased HT-mediated cytokine release compared to that in wild-type mice. Moreover, knockout of sphingosine kinase 1 completely or significantly abolished the cytokine generation inhibitory effects of atorvastatin (Figure 2). The cytokines induced by HT in SphK1^−/−^ mouse BAL in the absence or presence of atorvastatin were as follows: IL6 (SphK1^−/−^ Vehicle/HT vs. SphK1^−/−^ Atorva/HT, 180.50 ± 8.27 vs. 186.43 ± 42.62 pg/ml, *P* > 0.05, *n* = 5); KC (SphK1^−/−^Vehicle/HT vs. SphK1^−/−^ Atorva/HT, 156.83 ± 29.57 vs. 180.27 ± 69.14 pg/ml, *P* > 0.05, *n* = 5); MIP-1*α* (SphK1^−/−^ Vehicle/HT vs. SphK1^−/−^ Atorva/HT, 52.13 ± 23.47 vs. 28.95 ± 13.38 pg/ml, *P* > 0.05, *n* = 5); MIP-2 (SphK1^−/−^ Vehicle/HT vs. SphK1^−/−^ Atorva/HT, 90.82 ± 37.36 vs. 65.64 ± 14.72 pg/ml, *P* > 0.05, *n* = 5); MCP-1 (SphK1^−/−^ Vehicle/HT vs. SphK1^−/−^ Atorva/HT, 65.48 ± 20.81 vs. 60.17 ± 13.41 pg/ml, *P* > 0.05, *n* = 5), respectively. However, we failed to observe the same cytokine response to HT and the effect of pretreatment of atorvastatin, such as IL-1*β* (Figure 2).

### 3.3. Identification of Endothelial Cell Isolation from WT and SphK1^−/−^ Mice

**Figure 3:**
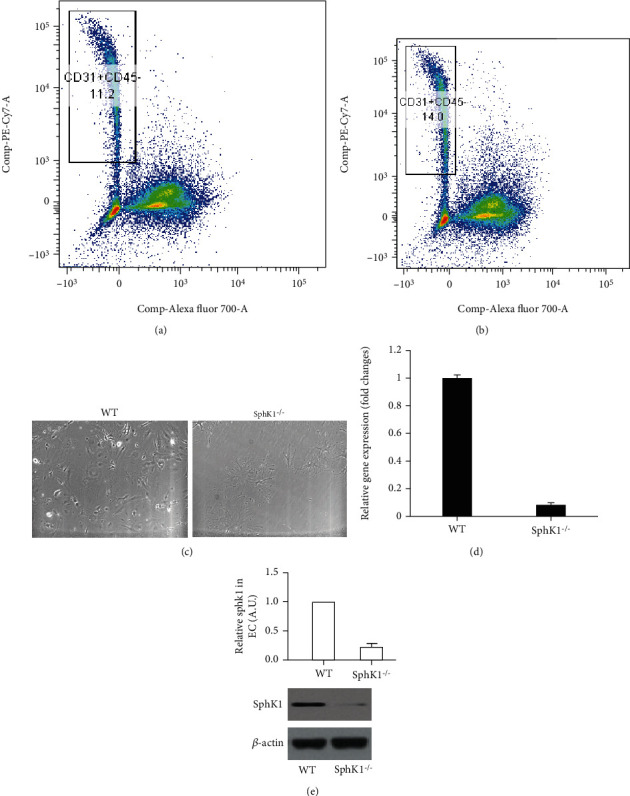
The endothelial cell isolation from WT and SphK1^−/−^ mouse lung and identification. The 8-week WT and SphK1^−/−^ mice were sacrificed, and lungs were harvested as described in Materials and Methods. (a) The endothelial cells of WT mouse lung were purified by flow cytometry (CD31^+^/CD45^−^). (b) The endothelial cells of SphK1^−/−^ mouse lung were isolated and purified (CD31^+^/CD45^−^). (c) The purified WT and SphK1^−/−^ endothelial cells were cultured on glass slides, and morphological properties were compared, including size, lamellipodia, and junctional properties. (d) qPCR and (e) western blot were performed to determine the sphingosine kinase 1 mRNA and protein level in purified endothelial cells.

To further confirm the role of sphingosine kinase 1 in atorvastatin-mediated anti-inflammatory effect, we then isolated the endothelial cells from WT and SphK1^−/−^ mouse lung. As shown in Figure 3(a), the endothelial cells were isolated (CD31+/CD45-) from WT mouse lung (SphK1^+/+^) and from SphK1^−/−^ mouse lung (SphK1^−/−^) by flow cytometry (Figures 3(a) and 3(b)). The isolated endothelial cells showed classic morphological properties of endothelial cells, such a triangle cellular body and two major lamellipodia. Interestingly, the isolated SphK1^−/−^ endothelial cells showed bigger and flatter cellular body with more lamellipodia (Figure 3(c)). After culture for several passages, the sphingosine kinase 1 mRNA and protein levels in WT and in SphK1^−/−^ endothelial cells were confirmed by qPCR (Figure 3(d)) and western blots (Figure 3(e)).

### 3.4. Sphingosine Kinase 1 Knockout Diminished the Atorvastatin-Mediated Anti-Inflammatory Effect in Mouse Endothelial Cells Responding to Cyclic Stretch

**Figure 4:**
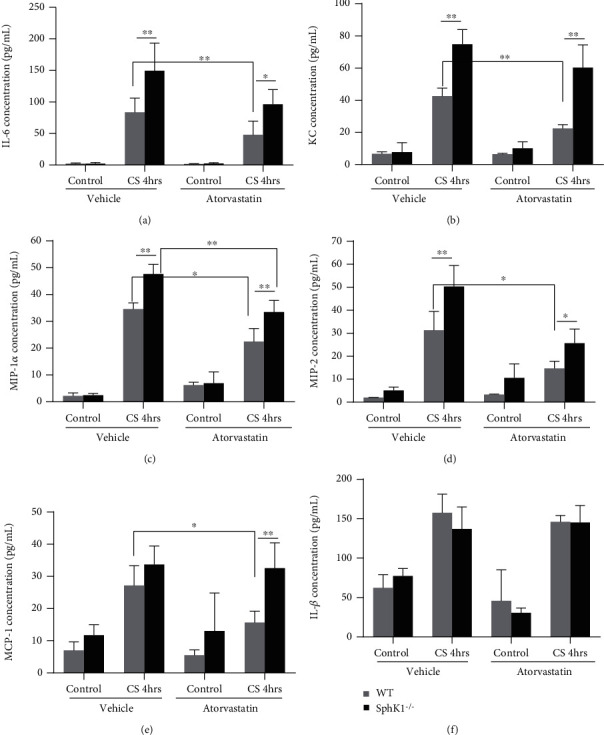
Atorvastatin attenuated cyclic stretch- (CS-) mediated cytokine generation from mouse-isolated endothelial cells, but SphK1 knockout reversed this effect. The isolated ECs were seeded on Flexcell plate and cyclic stretched (CS) (18% elongation, 0.5 Hz, 4 hours). After cyclic stretch, the medium was harvested for ELISA. CS significantly increased cytokine release in the WT endothelial medium. These cytokines include IL6, KC, MIP-1*α*, MIP-2, and MCP-1, but not IL-1*β*. The CS-mediated cytokine release in WT mouse endothelial medium was significantly attenuated by pretreatment of atorvastatin; CS-mediated cytokine concentrations were significantly higher in SphK1^−/−^ mouse endothelial medium than those in WT mice. Moreover, SphK1 knockout reversed the inhibitory effect of atorvastatin in cytokine generation in endothelial cells. (^∗^*P* < 0.05, ^∗∗^*P* < 0.01).

The isolated endothelial cells on Flexcell plates were cyclic stretched for 4 hours (18% elongation, 0.5 Hz), and the cytokines released from endothelial cells were measured by ELISA kits. As shown in Figure 4, the 4-hour cyclic stretch induced significantly higher cytokine generations in WT mouse endothelial cells than the static controls. Atorvastatin pretreatment significantly inhibited IL6 (WT Vehicle/CS vs. WT Atorva/CS 4 h, 83.73 ± 22.50 vs. 48.02 ± 21.76 pg/ml, *P* < 0.01, *n* = 5), KC (WT Vehicle/CS vs. WT Atorva/CS 4 h, 42.52 ± 5.05 vs. 22.42 ± 2.45 pg/ml, *P* < 0.01, *n* = 5), MIP-1*α* (WT Vehicle/CS vs. WT Atorva/CS 4 h, 34.58 ± 2.28 vs. 22.48 ± 4.84 pg/ml, *P* < 0.05, *n* = 5); MIP-2 (WT Vehicle/CS vs. WT Atorva/CS 4 h, 31.32 ± 8.13 vs. 14.67 ± 3.18 pg/ml, *P* < 0.05, *n* = 5); and MCP-1 (WT Vehicle/CS vs. WT Atorva/CS 4 h, 27.18 ± 6.16 vs. 15.69 ± 3.49 pg/ml, *P* < 0.05, *n* = 5) generation responding to cyclic stretch (Figure 4).

Similar with WT endothelial cells, CS also significantly increased cytokine release in SphK1^−/−^ endothelial cells compared to the static control. Moreover, SphK1 knockout significantly reversed the inflammation inhibitory effect of atorvastatin on cyclic stretch-mediated cytokine generation in mouse endothelial cells. Atorvastatin pretreatment did not significantly inhibit IL6 (SphK1^−/−^ Vehicle/CS vs. SphK1^−/−^ Atorva/CS 4 h, 149.0 ± 43.75 vs. 96.43 ± 23.08 pg/ml, *P* > 0.05, *n* = 5), KC (SphK1^−/−^ Vehicle/CS vs. SphK1^−/−^Atorva/CS 4 h, 74.82 ± 9.14 vs. 60.26 ± 14.22 pg/ml, *P* > 0.05, *n* = 5), and MCP-1 (SphK1^−/−^ Vehicle/CS vs. SphK1^−/−^ Atorva/CS 4 h, 33.73 ± 5.76 vs. 32.57 ± 7.91 pg/ml, *P* > 0.05, *n* = 5) generation responding to cyclic stretch in SphK^−/−^ EC.

Notably, sphingosine kinase 1 knockout significantly but not completely reversed the inhibitory effect of atorvastatin on MIP-1*α* (SphK1^−/−^ Vehicle/CS vs. SphK1^−/−^ Atorva/CS 4 h, 47.68 ± 3.61 vs. 33.46 ± 4.39 pg/ml, *P* < 0.01, *n* = 5) and MIP-2 (SphK1^−/−^ Vehicle/CS vs. SphK1^−/−^ Atorva/CS 4 h, 50.33 ± 9.11 vs. 25.64 ± 6.21 pg/ml, *P* < 0.05, *n* = 5) generation (Figure 4).

### 3.5. Sphingosine Kinase 1 Knockout Turned Over Atorvastatin-Mediated MAPK Phosphorylation Inhibitory Effects on Cyclic Stretch Model

**Figure 5:**
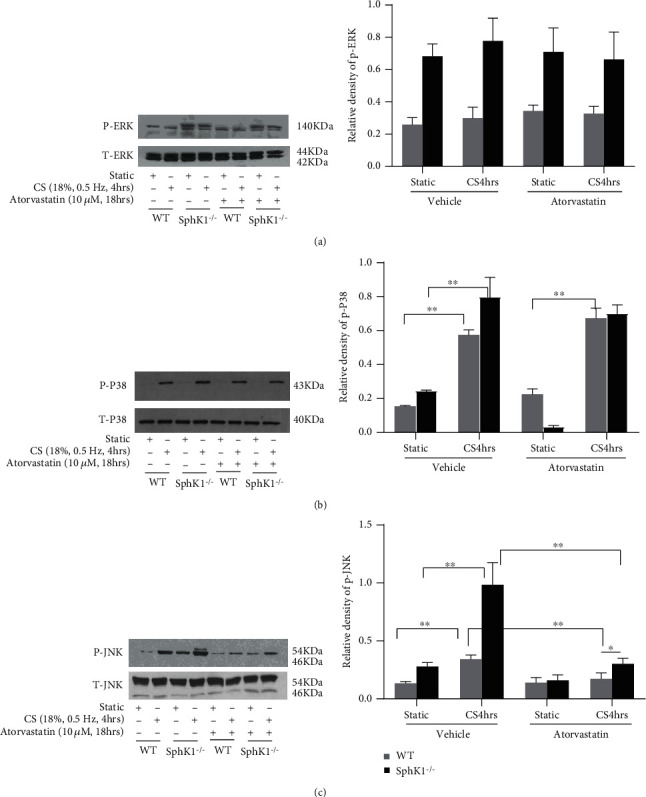
Atorvastatin attenuated cyclic stretch- (CS-) mediated MAPK phosphorylation, and SphK1 knockout reversed this effect. After CS finished at indicated time, the cellular lysates were harvested for western blotting. Compared to the static control, CS significantly induced (b) P38 and (c) JNK phosphorylation in mouse WT ECs. The MAPK phosphorylation was significantly attenuated by pretreatment of atorvastatin. Compared with WT endothelial cells, SphK1^−/−^ cells showed enhanced CS-mediated P38 and JNK phosphorylation. Moreover, CS-mediated P38 and JNK phosphorylation levels in SphK1^−/−^ ECs were significantly higher than those in WT ECs after treatment of atorvastatin. (^∗^*P* < 0.05, ^∗∗^*P* < 0.01).

We next investigated the atorvastatin-regulated potential signal pathway which mediates the mechanic stretch-induced inflammatory response. Previous studies showed that MAP kinase including ERK, P38, JNK phosphorylation, or other modifications are changed by the mechanic stretch accompanying inflammatory response. 18% elongation 0.5 Hz cyclic stretch induced P38 and JNK phosphorylation increase in WT mouse endothelial cells, and atorvastatin significantly inhibited MAPK phosphorylation induction (Figure 5).

Similar with WT endothelial cells, 18% elongation 0.5 Hz cyclic stretch also induced P38 and JNK phosphorylation increase in SphK1^−/−^ mouse endothelial cells. Pretreatment of atorvastatin significantly inhibited JNK but not ERK and P38 phosphorylation (Figure 5).

### 3.6. Sphingosine Kinase 1 Knockout-Mediated Cytokine Generation Enhancement in Endothelial Cells May Be Regulated by JNK and P38

**Figure 6:**
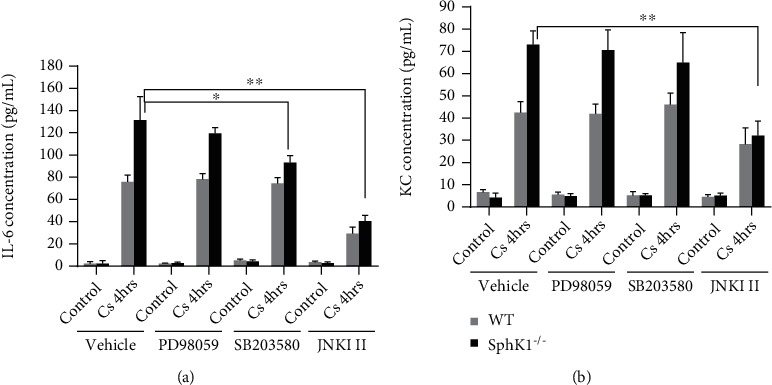
JNK plays a critical role in sphingosine kinase 1-mediated inflammatory response to cyclic stretch. The isolated ECs were seeded on Flexcell plates overnight in the presence of the indicated inhibitors. After cyclic stretching (CS) (18% elongation, 0.5 Hz, 4 hours) as described in Figure 4, the medium was harvested for ELISA. (a) IL6 and KC assay showed that SphK1 knockout significantly enhanced CS-mediated IL6 and KC generation. The SphK1 knockout-mediated IL6 generation enhancement was not significantly abolished by PD98059 but partially by SB203580 and completely by JNK inhibitor II. (b) The SphK1 knockout-mediated KC generation enhancement was not significantly abolished by PD98059 and SB203580, but completely by JNK inhibitor II (^∗^*P* < 0.05, ^∗∗^*P* < 0.01).

To further confirm the regulation of sphingosine kinase 1 knockout on MAPK, the isolated mouse endothelial cells were cyclic stretched in the presence of ERK, JNK, and P38 inhibitors. After 4-hour cyclic stretch, the cytokines in medium were measured. As shown in Figure 6, sphingosine kinase 1 knockout induced significant IL6 and KC generation in SphK1^−/−^ endothelial cells comparing to WT endothelial cells. The IL6 generation induction in SphK1^−/−^ endothelial cells was inhibited by JNK inhibitor and partially inhibited by SB203580 (P38 inhibitor) but not by PD58059 (ERK inhibitor) (Figure 6(a)). The CS-mediated KC generation in SphK1^−/−^ endothelial cells showed similar trends (Figure 6(b)).

### 3.7. Sphingosine Kinase 1 Plays a Critical Role in Atorvastatin-Mediated Endothelial Junction Integrity Enhancement

**Figure 7:**
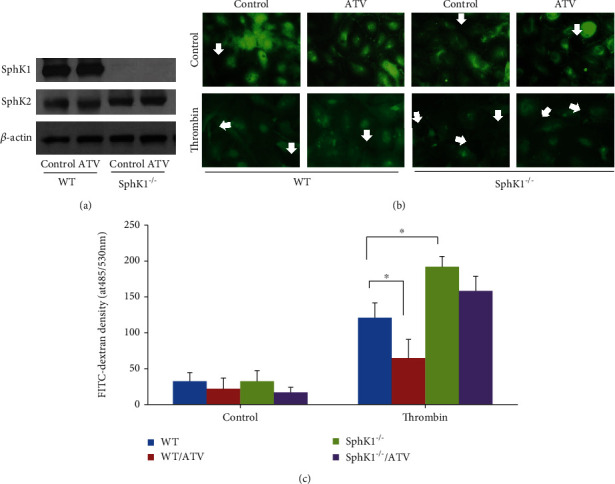
Atorvastatin did not change SphK1 expression in mouse EC but enhance EC junction integrity. (a) The ECs isolated from WT and SphK1^−/−^ mice were cultured in 6-well plates. The protein levels of SphK1 and SphK2 were determined by western blotting. (b) The ECs isolated from WT or SphK1^−/−^ mice were cultured on glass slides in a 12-well plate. After treatment with 10 *μ*M atorvastatin for 16 hours, the cells were challenged by 1 U/ml thrombin for 1 hour. Immunostaining was performed with VE-cadherin antibody as described in Materials and Methods. (c) Monolayer endothelial cells were cultured on transwell inserts and treated with 10 *μ*M atorvastatin for 16 hours. The next day, the cells were treated with 1 U/ml thrombin for 1 hour in the presence of FITC-dextran. The FITC-dextran fluorescent density in the bottom chamber was measured at 485/538 nm (^∗^*P* < 0.05).

As pretreatment of atorvastatin decreased both protein and cellular filtration on the in vivo model, we then investigated the importance of atorvastatin on the endothelial barrier integrity against pathogenic challenge like thrombin. We first tested whether atorvastatin changed SphK1 expression in mouse endothelial cells. As shown in Figure 7(a), atorvastatin did not change SphK1 in WT and SphK1^−/−^ endothelial cells (upper panel). Atorvastatin also did not change another isotype of sphingosine kinase, SphK2 (middle panel).

We next investigated whether SphK1 is involved in the endothelial barrier integrity enhancement. Immunostaining showed that VE-cadherin mostly located in the cytosolic plasma and a few small gaps existed between WT endothelial cells. Thrombin challenge increased both the size and the numbers of gaps between endothelial cells (Figure 7(b) upper panel). Atorvastatin treatment caused VE-cadherin translocating to cellular membrane accompanying enhancement of endothelial junction integrity at both control and thrombin challenge condition (Figure 7(b) upper panel); However, the atorvastatin-mediated EC VE-cadherin junction integrity enhancement effect significantly decreased in SphK1^−/−^ endothelial cells (Figure 7(b) right side).

To further confirm the role of SphK1 in atorvastatin-mediated endothelial junction integrity enhancement effect, the permeabilities of monolayer WT and SphK1^−/−^ endothelial cells in transwell plates were compared at both control and thrombin-challenging conditions (Figure 7(c)). Thrombin challenge caused FITC-dextran permeability significantly increased at WT endothelial cells compared to control. Additionally, the thrombin-mediated FITC-dextran permeability was attenuated by pretreatment by atorvastatin (Figure 7(c)). Compared to WT endothelial cells, thrombin-challenged SphK1^−/−^ endothelial cells had significantly higher FITC-dextran permeability. Moreover, this permeability increase was not significantly reversed by atorvastatin (Figure 7(c)).

## 4. Discussion

**Figure 8:**
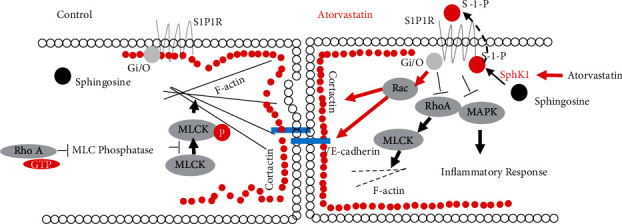
Schematic description of the potential role of SphK1 in atorvastatin-mediated anti-inflammatory effect. Under control condition, sphingosine exists as an inactive form and there is balance between the stress fiber system and the cortactin ring system. When atorvastatin is added, stress fiber is inactivated, the cortical ring is enhanced, and the VE-cadherin junction is tightened. Meanwhile, atorvastatin inhibits the MAPK signal pathway and attenuates inflammatory responses. Sphingosine kinase 1 is a mediator in these processes.

Based on the murine high tidal volume ventilation model and cellular models, the importance of sphingosine kinase in atorvastatin-mediated anti-inflammatory effects was investigated. Atorvastatin significantly attenuated VILI-mediated protein and cell infiltration as well as cytokine release on in vivo and cellular model of WT mice. Knockout SphK1 diminished the atorvastatin-mediated inflammatory inhibitory effects. Signal pathway assay showed that SphK1-mediated anti-inflammatory effect was partially through the JNK pathway. In addition, endothelial junction studies indicated that SphK1 plays a central role in endothelial junction reorganization and integrity maintenance, which also partially contributes to the anti-inflammatory effect of atorvastatin (Figure 8).

Mechanical stress-mediated inflammatory response has its own unique properties, such as sever junctional damage and in homogenous lung injury due to uneven pleural pressure [38, 39]. Long-term side effects include COPD [40]. The molecular mechanism of mechanical ventilation includes barotrauma, volutrauma, atelectrauma, and biotrauma [41]. Among all the mechanism basis of VILI, endothelial barrier is the most sensitive and plays a central role in the process of inflammatory response to VILI. The involvements of endothelium in VILI include (1) increased lung vascular permeability due to loss of endothelial cell barrier integrity, (2) mechanical stress-induced inflammatory cytokine release and cell infiltration, and (3) genetic and epigenetic regulation of critical target genes such as junction protein and signal proteins that are involved in VILI response [17]. The anti-inflammatory effects of statins have been widely investigated on various models [6, 42–44]. These anti-inflammatory effects include decreasing proinflammatory cytokine generation, enhancing endothelial cell junction integrity, decreasing ROS and chemokine generation, decreasing lipid oxidation, and inflammation [31, 45]. The beneficial effects of statins on mechanical stress-mediated acute lung injury have been relatively poorly understood [43]. In current studies, we identified the atorvastatin-mediated anti-inflammatory mechanisms on cellular model and VILI model. The inhibitory effect of atorvastatin was mediated by inhibition of MAPK activity, which is similar to other statins [32, 46]. Moreover, sphingosine kinase 1 also contributes to atorvastatin-mediated endothelial junction integrity enhancement [47, 48].

SphK1 is involved in many critical cellular activities [49, 50]. Sphingosine-1-phosphate, a product of SphK1 and sphingosine, has shown enhancing endothelial junction. By binding with the EDG receptors, S-1-P activates Rac GTPase, causes cytoskeleton reorganization, and improves endothelial junction tightness [51]. Interestingly, pharmaceutical inhibitor studies demonstrate that the MAPK signal pathway is not involved in this process [52]. Knockout SphK1 resulted in the increase of LPS- or PAR-induced edema in mouse lung and cellular barrier permeability in cellular model [27]. Recent studies indicated that SphK1 stabilizes JNK and inhibits the interaction of JNK and JNK-interacting protein 3 (JIP3), further abrogating the activation of NADPH oxidase and NF-*κ*B activation [28]. In addition, SphK1 also plays a protective role in LPS-mediated inflammatory response in the central nervous system as well as other inflammatory responses [53, 54]. The results in the current study show that SphK1 plays a critical role in atorvastatin-mediated protective effects against high tidal volume ventilation-induced lung injury (VILI). One of the major mechanisms of atorvastatin may mediate MAPK activity inhibition during the inflammatory response. Knockout of the sphingosine kinase 1 abolished the inhibition effect of atorvastatin on MAPK activity indicating that sphingosine kinase 1 plays a vital role in atorvastatin-mediated MAPK inactivation. These are also supported by VILI-mediated cytokine releases from SphK1^−/−^ cellular and mice.

Another important contribution of sphingosine kinase 1 in atorvastatin-mediated anti-inflammatory effects is the enhancement of endothelial junction integrity. Endothelial junction includes many components and is regulated by multiple dynamic systems. So far, endothelial junction includes atypical tight junction, which consists of ZO-1, occluding, claudins, JAM, etc. Another major junction is adherens junction, which consists of VE-cadherin, *α*-catenin, *β*-catenin, etc. Moreover, endothelial junction integrity also depends on cytoskeleton protein such as F-actin and MLCK as they associate with and tightly regulate junctional proteins. When stimuli like LPS or thrombin bind with their receptor on endothelial cells, the relative signal pathways regulate cytoskeleton rearrangement. High tidal volume ventilation may activate cytoskeleton system by modulating mechanical receptors. The consequence of these challenges to the lung will present as edema and inflammatory cell infiltration. SphK1 turns sphingosine into an active form, sphingosine-1-phosphate (S-1-P). S-1-P in turn regulates the VE-cadherin junction and cortical actin ring [55]. Consistently, endothelial junction imaging and permeability assays indicate that sphingosine kinase 1 is a critical mediator of atorvastatin-mediated endothelial junction integrity enhancement.

In summary, the regulatory mechanism of atorvastatin on anti-inflammatory response to mechanical stress has been investigated. By inactivation of MAPK in endothelial cells and enhancement of endothelial junction integrity, atorvastatin attenuates VILI-mediated inflammatory response. This anti-inflammatory response of atorvastatin was abolished by sphingosine kinase 1 knockout. These findings in the current study open a new field for us to explore the new medicine for acute lung injury, such as high tidal volume ventilation-mediated lung injury.

## Data Availability

The data (Excel) used to support the findings of this study are available from the corresponding author upon request.
